# Design and Evaluation of DentAll Mobile Software for Dental Education 

**DOI:** 10.30476/JAMP.2021.90771.1423

**Published:** 2021-10

**Authors:** PARISA JALALI, ZEINALABEDIN GHOLIZADEH, MINOU KOUH SOLTANI, MARYAM KOUHSOLTANI

**Affiliations:** 1 Dental and Periodontal Research Center, Tabriz University of Medical Sciences, Tabriz, Iran; 2 Mianeh Technical and Engineering Faculty, University of Tabriz, Tabriz, Iran; 3 Department of Oral and Maxillofacial Pathology, Faculty of Dentistry, Tabriz University of Medical sciences, Tabriz, Iran

**Keywords:** Dentistry, Education, Learning, Software

## Abstract

**Introduction:**

The use of new learning methods to improve the educational processes is inevitable in the field of dentistry. Due to the positive effects of mobile learning on the students’ learning and skills, we decided to design a software in the required areas. Considerations of systemic diseases, medications, emergencies, prophylaxis, tests interpretation, lesions diagnosis, cephalometric, and cast analysis were the topics required.

**Methods:**

This semi-experimental study was carried out in Faculty of Dentistry, Tabriz University of Medical Sciences during 2020. DentAll software was developed using Android Studio software. It is an educational and decision-making support system. The satisfaction and perceptions of 10 professors, 5 post-graduate students, and 30 senior dental students about the designed software were assessed using a questionnaire based on a 5-part Likert scale. The senior students were invited and the pre-test was taken to assess the students' knowledge; then, the designed software was provided by the administrator. At the end of the process, the post-test was taken. Paired samples t-test was used to compare the results of the pre-test and post-test. The data were analyzed using the Statistical Package for Social Sciences (SPSS) 17.0. The significance level was established at a P < 0.05.

**Results:**

The mean scores of the pre-test and post-test were 16.5 ± 1.49 and 18.2 ± 0.99, respectively. The statistical analysis showed that the difference between the mean scores of the pre-test and post-test was statistically significant (p< 0.001). 46.7% of the professors and post-graduate students expressed their overall satisfaction as very high, 46.7% as high, and 6.7% as average. Furthermore, 50% of the students expressed their overall satisfaction as very high, 36.7% as high, and 13.3% as moderate.

**Conclusion:**

The designed comprehensive software satisfied the students and the professors who used it and the students' scores increased after use. This software had capabilities such as easy access to the resources, learning anytime and anywhere, increasing the students' interest and motivation, etc. from the point of view of the commentators. To the best of our knowledge, this comprehensive educational aid has not been designed previously and it was effective in improving the students' learning, scientific knowledge and treatment planning.

## Introduction

In recent decades, new communication methods make the learning process possible outside the classroom. Students, as self- directed learners, use information and communication technology. One of the communication and information resources is mobile learning, which is especially popular among medical students ( 1 ) and the students' tendency in using smartphones for learning is increasing ( 2 ). This learning tool can increase the students' enthusiasm to learn without time and space constraints and provide an opportunity to learn in spare times ( 3 ).

Increasing access to appropriate hardware and software for e-learning has opened a new horizon for educational institutions. It seems that the use of these facilities for education provides the context of ideal learning that is referred to as the criterion of education quality, e.g. active learning, continuous learning, etc. ( 4 - 5 ).

Medical students often use mobile phones and software for tasks such as information management, time management, health record maintenance and access, communications and consulting, reference and information gathering, clinical decision-making e.g. clinical decision support systems, differential diagnosis, patient monitoring, patient care, medical education, and training ( 6 - 7 ). The medical students' attitudes toward the use of mobile devices and software are quite positive ( 8 - 9 ). The most important benefits of mobile learning are increasing the students' learning motivation, self-directed learning, convenience, efficiency, organization, and flexibility ( 7 ).

Many efforts have been made to design and evaluate the educational software in the medicine and dentistry. De Sena at el. conducted a study to investigate the effect of educational software on learning the skin flap surgery. They found that the skill scores of students who used the software were higher than the group who were trained in the traditional way ( 10 ). Khanal at el. showed that the use of virtual world based on educational simulation of cardiopulmonary resuscitation is as effective on the learning process as the traditional face-to-face method ( 11 ). Safdari et al. studied the design of the educational software of cardiopulmonary resuscitation simulator and its effect on students’ satisfaction. The results showed that the level of the students' satisfaction about the software was good ( 12 ). Deshpande et al. designed a prosthodontics software and evaluated the students' satisfaction and the effect of software on the students' clinical decision-making skills; then, they observed that the software had improved clinical reasoning and decision-making skills ( 13 ). Gilavand et al. designed an educational software for considerations for systemic patients, so that on clicking on the name of each disease, a page would open in which potential problems of the disease in dental treatment, its manifestations and symptoms, and the solution to prevent systemic problems during dental treatment were shown ( 14 ).

Studies show widespread use of mobile phones and software in medical education, and this new technology offers a potential to improve the learning process and treatment plans ( 7 ). The most practical aspect of educational software is that students have easy access to content and save (manage) time ( 15 ).

The enormous content and rapid growth of information in dentistry have encouraged the use of various technologies to improve learning process. So far, several software has been designed for oral ulcers and systemic diseases, but there is no comprehensive software in the field of dentistry. 

Today, due to the advancement of medical science in controlling many diseases and increasing life expectancy, more people continue to live despite systemic diseases. For dental treatment of these patients, dentists should make changes in their treatment plan to provide a safe treatment; the consequences of lack of sufficient knowledge in this area are irreparable. In addition to diagnosis and provision of a suitable treatment plan, dentists must have sufficient ability. In order to deal with medical emergencies that require timely diagnosis and treatment, they must, as health care providers, prepare themselves for the control of medical emergencies and have enough knowledge about drug interactions and side effects of medications ( 16 ). Also, one of the educational problems is the lack of sufficient skills in the field of transcription ( 17 ). Therefore, education and easy access to how to prescribe common drugs are welcomed by students.

Cephalometric analysis for orthodontic treatment plan is time consuming in the way of tracing on the paper and measuring angles with a conveyor; and a software is valuable for saving time and accurate analysis.

Early detection of oral lesions helps the dentists to prevent the progression of the lesion with appropriate planning. Various factors can interfere with the diagnosis of lesions. One of these factors is the similar views of the diseases that make the dental students forget or confuse some when faced with a specific lesion. Therefore, it is necessary to use a decision support system with the ability to process information, so we decided to add a decision support to the software and evaluate its efficiency.

Considering the general use of mobile phones among students and the positive results of providing education based on it and the prevalence of corona pandemic nowadays that has highly influenced educational methods, we decided to design DentAll software and assess the students' learning and evaluate the satisfaction and perception of professors and students about this software. We hope the results of this study help in raising the students' knowledge and learning and better management of patients.

## Methods

This semi-experimental study was carried out in Faculty of Dentistry, Tabriz University of Medical Sciences during 2020. The study was performed in three consecutive steps: design, implementation and evaluation of DentAll software. The following aims were considered in designing the software:

1- Consideration for systemic patients and medical emergencies in dentistry
2- Prescription and use of common medications, prophylaxis, and interpretation of laboratory tests
3- Cephalometric analysis
4- Decision-making aid for differential diagnosis of hard tissue lesions and diagnosis of the pulp status.

### Software Design

#### The software environment

The DentAll software was developed using Android Studio software and can be run in different platforms with Android environment. For developing the application, first the algorithm was derived adopting the contents from the latest manuscripts and the latest editions of dental reference books such as Little and *Falace's Dental Management* of the Medically Compromised Patient, Burket’s Oral Medicine, Peterson’s Oral and Maxillofacial Surgery, White and Pharoah’s Oral Radiology, Proffit’s Orthodontics, and Torabinejad’s Endodontics Principles and Practice. After collecting the data from dental sources, it was validated by 10 expert professors. Then, the algorithms were coded into Android Studio. This software was adopted due to its accuracy, faster and easier testing and programming capability. The DentAll application size is 4.3 MB.

DentAll is an educational and decision making support system including the main sections described in Table 1. In each section, the software processes the entered information step by step in a logical order, and the final results are represented to the user by it. After coding the software, the professors and students tested it experimentally, and the problems were recognized and solved.

**Table 1:** Software structure.

Screen No.	Content
Screen 1	Home screen
Screen 2	The first page contains topics e.g. dental considerations in systemic patients, dental medications, medical emergencies in dentistry, prophylaxis, interpretation of laboratory tests, diagnosis and treatment plan.
Screen 3	Selecting the "Dental Considerations in Systemic Patients" option opens a page that contains a list of systemic diseases e.g. diabetes, hypertension, etc., and the user can access dental considerations by selecting each.
Screen 4	Selecting "Dental Medications" option opens a page that contains a list of the most commonly used drug types in dentistry, e.g. antibiotics, antifungal and antiviral drugs, dental anesthetics, nonsteroidal and opioid analgesics, mouthwashes, sedatives and antixerostomia. By selecting each group, the drugs are displayed; and by selecting the desired drug, the drug form, dosage, considerations and how to prescribe it are shown.
Screen 5	In the case of "Anesthetics", the maximum number of carpules for each patient is calculated by entering the patient's weight.
Screen 6	Selecting the "Medical Emergency in Dentistry" option displays a list of medical emergencies that may occur in the dental office, such as hypoglycemia, vasovagal, asthma, etc. By selecting any of the options, the necessary actions in that case are shown.
Screen 7	Selecting the "Prophylaxis" option displays information about prophylaxis conditions and medications as well as their dosage.
Screen 8	By selecting the "Interpretation of Laboratory Tests" option, a list of routine tests is displayed e.g. FBS, HBA1c, RBC, CBC, PT, PTT, ALT, AST, etc., and by selecting each, explanations are displayed.
Screen 9	In the "Endodontic Diagnosis and treatment plan" section, the user selects the options based on the patient's chief complaint, the examination, the signs and the symptoms; and the diagnosis is made in terms of pulpal and periodontal status and the treatment plan is suggested.
Screen 10	In the part of "Differentiation between Pulpal and Periodontal Disease" section, the user chooses the clinical and radiographic features to diagnose.
Screen 11	In the "differential diagnosis of hard tissue lesions" section, the user chooses options such as the location, the shape, the extent, the internal structure and the borders of the lesion as well as the clinical signs, and then reach the most likely differential diagnoses is made.
Screen 12	In the "Cephalometric Analysis" section, there is a lateral cephalometric image in the background with point selection tips. The user uploads the desired image from the gallery. To determine the desired point, at first that point must be selected from the list of points, then the desired area of the image, in normal or magnified size, and finally the desired analysis is performed by pressing the draw key.
Screen 13	In the "Caste Analysis" section, the user uploads the photo of the cast from the axial view and selects the desired points in order to space analysis.
Screen 14	In the "Subtraction" section, by uploading the image of before and after the treatment and adapting the desired location, the changes caused by the treatment are visible by image analysis.

#### The software content

 When the program is run, the first page contains topics such as dental considerations of systemic patients, dental medications, medical emergencies in dentistry, prophylaxis, interpretation of laboratory tests, and diagnosis and treatment plan; the user can access information by selecting each. For example, the user achieves the relevant considerations by selecting the desired disease. By selecting the desired medical emergency, necessary measures are displayed. Also, by selecting the desired drug from the drug applications section, drug considerations are displayed. In the case of anesthetics including common dental anesthetics, e.g. Articain, Lidocaine, Mepivacaine, Prilcaine, the maximum number of carpules for the patient is calculated by entering the patient's weight. 

**Figure 1:**
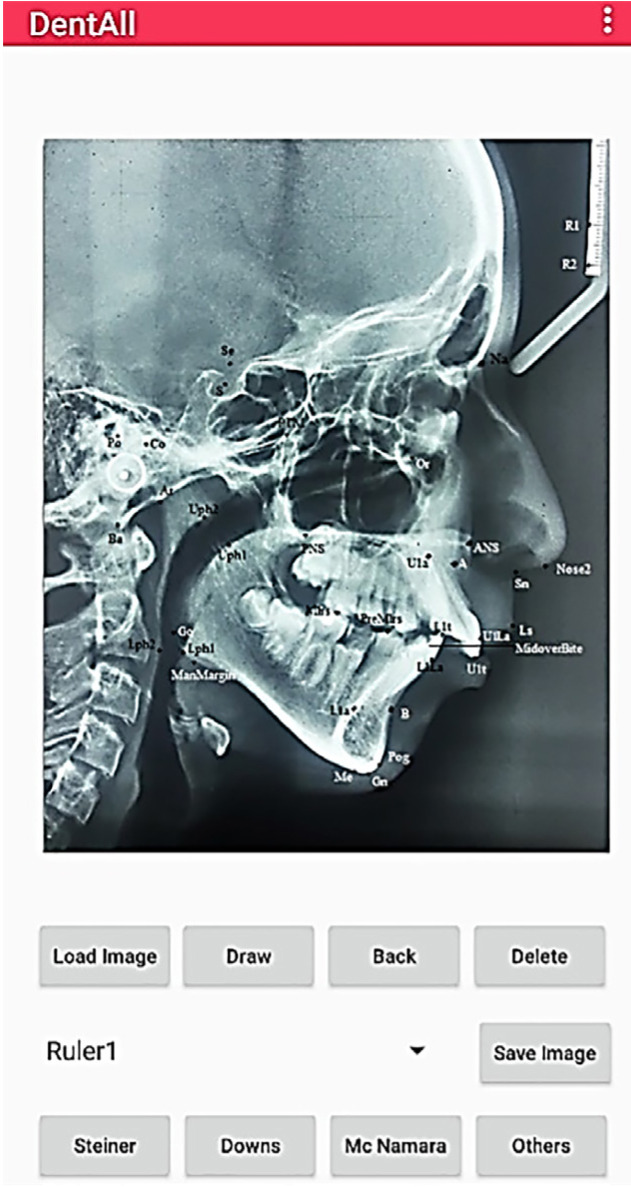
The cephalometric analysis screen

In prophylaxis and interpretation of the laboratory tests sections, by selecting each topic, explanations are displayed. In the diagnosis and treatment plan sections, by selecting the options based on clinical signs and symptoms and radiographic features, the software helps the user diagnose the dental pulpal conditions )such as normal, symptomatic irreversible pulpitis, etc.) and differentiate it from periodontal diseases or differential diagnosis of hard tissue lesions. Furthermore, this software includes cephalometric and space analyses and subtraction. As shown in Table 1, the software consists of multiple screens, and some screens are shown in Figures 1 2- 3.

**Figure 2:**
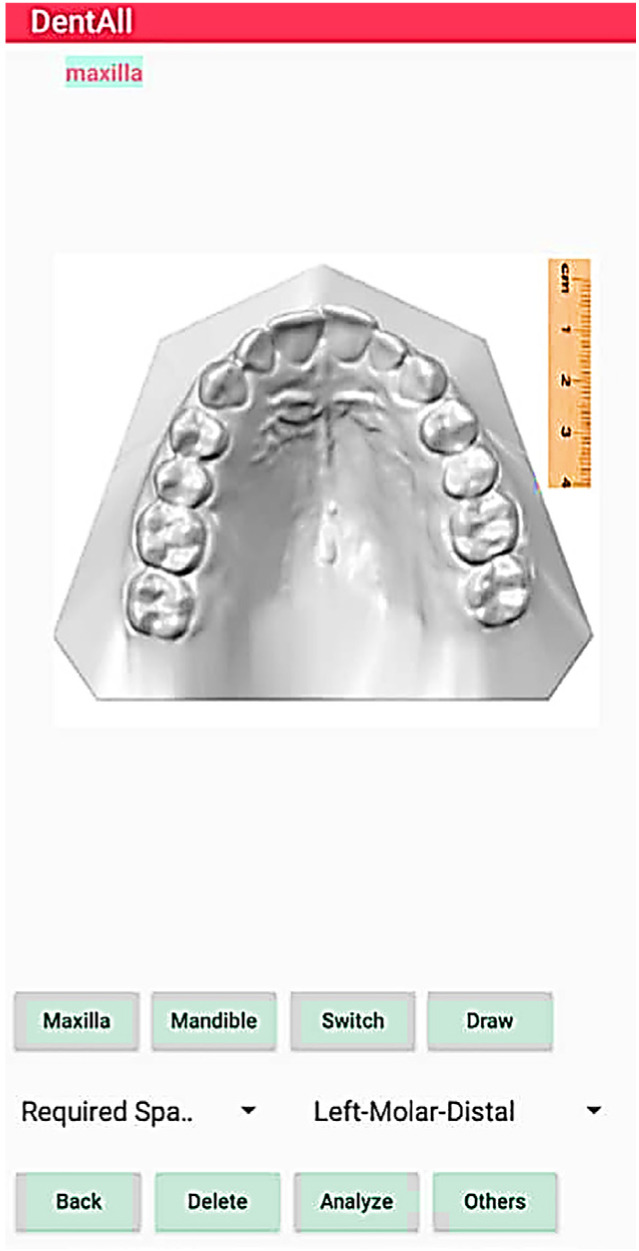
The cast analysis screen

**Figure 3:**
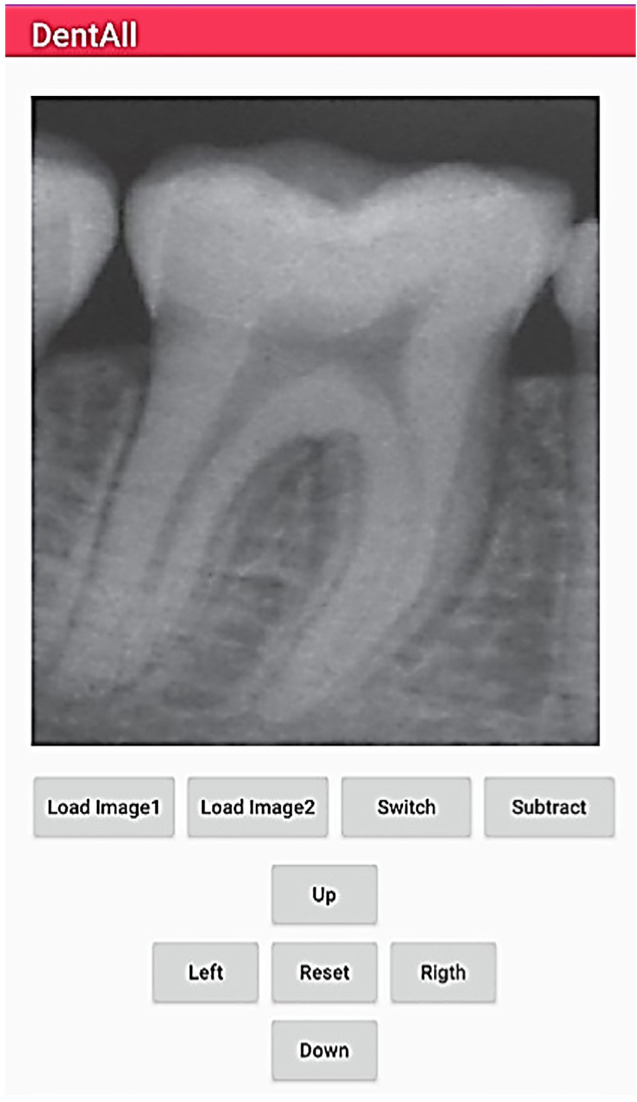
The subtraction section

#### Implementation and evaluation

30 senior dental students were invited to participate in the assessment. The inclusion criteria for the students were their willingness to participate in the study, availability of an Android-based smartphone, and grade point average more than 17 to ensure that they would definitely use the software. The pre-test was performed using 20 questions to assess the students’ knowledge. Then, they were provided with DentAll software by the administrator to study the educational contents of the software. Finally, the post-test was given. 

Furthermore, the students and the professors’ satisfaction and perceptions about the designed software was assessed using a questionnaire based on a 5-part Likert scale. The questionnaire consisted of 5 main parts: software educational content, learning and effectiveness, innovation, application, design, and the overall satisfaction. The Kirk Patrick model was used. The questionnaires were filled by 10 professors and 5 post-graduate students. The mentioned comments were collected and applied in order to improve the software. 

#### Statistical analysis

The results were expressed as percentage, frequency and Mean±SD. Paired t-test was used to compare the results of the pre-test and the post-test. The data were analyzed using the Statistical Package for Social Sciences (SPSS) 17.0 (SPSS, Chicago, IL). A *P value of* < 0.05 was considered as significant. 

#### Ethical Consideration

This manuscript was taken from a scholarly educational process and awarded in 22th national Shahid Motahhari Educational Festival. All ethical issues including the confidentiality of information, opinions and ratings have been considered.

## Results

**Figure 4:**
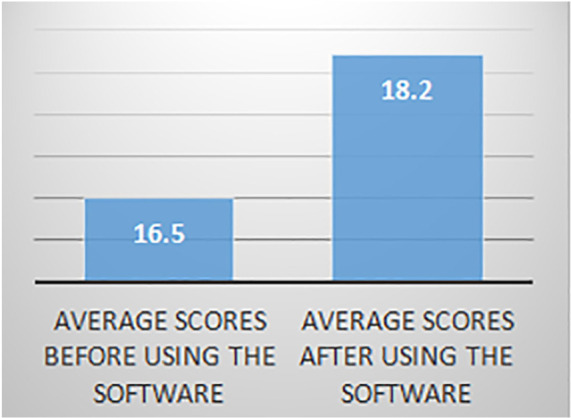
Results of the pre-test and the post-test

**Figure 5:**
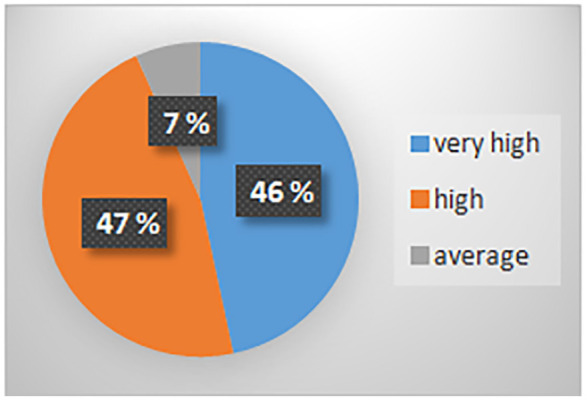
Overall satisfaction of the professors with the software

**Figure 6:**
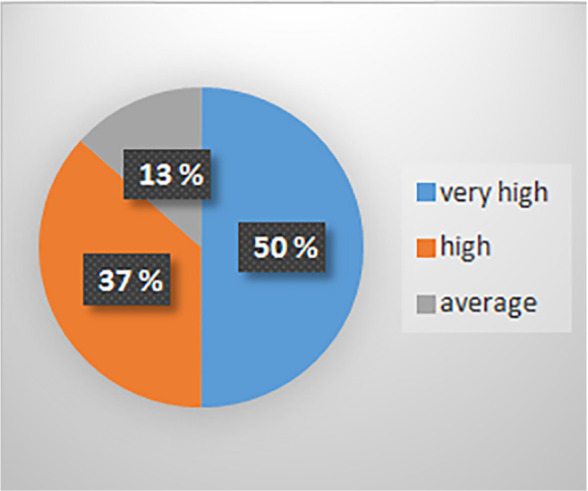
Overall satisfaction of the students with the software

This study included 30 senior dental students. Their age range was 22-25 years old. Out of 30 students, 19 were female and 11 were male. The mean scores of the pre-test and post-test were 16.5±1.49 and 18.2±0.99, respectively. Paired samples t-test was used for statistical analysis ( Figure 4). The results showed that the difference between the mean scores of the pre-test and post-test was statistically significant (P<0.001). 46.6% of the professors and post-graduate students expressed their overall satisfaction with the software as very high, 46.7% as high, and 6.7% as average ( Figure 5). Furthermore, 50% of the students expressed their overall satisfaction about the software as very high, 36.7% as high, and 13.3% as moderate ( Figure 6).

A selection of most important questions and the agreement percentage of the professors and the students are shown in Table 2.

**Table 2:** Samples of the most important questions of the questionnaire and the agreement percentage.

Questions	Professors' agreement	Students' agreement
The content of the software is appropriate for the educational needs of the students.	100%	90%
The scientific quality of the educational content of the software is appropriate.	86.6%	83.4%
Providing content in the form of educational software is useful in continuous students' learning.	93.3%	76.6%
The software helps the students to master the topics.	80%	80%
The existence of such a software as an educational aid helps to improve the clinical performance of the students.	75%	90%
Presenting the content in the form of educational software is useful in increasing the students' interest and motivation to learn.	80%	76.6%
This educational software is useful for the students after graduation.	100%	96.7%

Achievements and capabilities of the software according to the satisfaction survey were: easy access to the resources, possibility of learning anytime and anywhere, increase in the students' interest and motivation in learning, increase in the information maintenance in long-term memory by constantly memorizing the content, time management, enhancement of the students' learning about the considerations of systemic patients and medical emergencies in dentistry, prescription and use of drugs, prophylaxis and interpretation of laboratory tests, ease of orthodontic analysis without any need for a protractor and drawing on a paper and time saving.

## Discussion

According to the results of this study, the designed smartphone software improved the students' knowledge, increased their scores, and satisfied the professors and the students. Therefore, smartphone software programs can be used for educational purposes. Since the introduction of smartphones in the market, its use in education has developed. Studies show that the use of personal digital devices by medical students is well accepted, and it is useful in learning ( 18 , 19 ); actually, the tendency to use smartphones in students for learning purposes is increasing ( 20 ). Wallace et al. reported that not only 85% of medical students use mobile phones daily, but they also expect to use more of this technology to learn more; 77% of the study participants were using at least one software for learning. They had a tutorial installed on their mobile phone and used it regularly ( 7 ).

The use of educational software in dentistry is novel. Only few educational software have been developed with the aim of teaching topics related to dentistry. According to the results of this educational process, the designed software can improve the students’ knowledge in the desired field and the mobile phones are useful for educational purposes. According to previous studies, the most popular educational software used by medical students is the one related to diagnosis and treatment planning ( 17 - 20 ). Students express their need for more software in order to be guided in clinical decision making ( 21 - 22 ).

The effectiveness of software on education has been proven by many authors, e.g. Chang, et al ( 23 ). Numerous studies have been conducted to evaluate the effect of using mobile software on improving the performance and skill score of the students, indicating its positive and effective results ( 24 ). Chase and colleagues concluded that the use of mobile devices are very effective in education; most students tend to use educational software in their spare time, and these programs do not have to be forced to use ( 25 ). Golenhofen et al. studied the effect of App-eMed software on learning anatomy lessons. The results showed that more than 70% of the students used this software, and those who had higher scores had benefited more from this software compared with the students with lower scores ( 26 ).

Mladenovic et al. designed a software about dental traumatic injury to motivate the students to learn more during the corona pandemic holiday in Serbia. Thirty-one final year medical students were assessed; it was concluded that more than 90% of the students were satisfied and the software provided faster access to clinical content ( 27 ). Gilavand et al. did a research with the aim of evaluating the effect of educational software on raising the students' knowledge of systemic patients' considerations. The user used this software to observe the dental considerations and potential problems of the systemic diseases in dental treatments, manifestations of the disease, and oral symptoms. The results of this study indicated an increase in the scores of the group that used the software; this is consistent with the results of this study ( 14 ).

In a systematic review study on the educational impact of mobile learning in medical students, 21 experimental studies were extracted from articles published from 2007 to 2017. The results showed that medical students had a positive attitude towards mobile learning. In addition, the implementation of mobile learning program in medical education programs can increase the potential educational benefits and clinical competence along with knowledge and attitudes. The results showed that mobile learning strategy in medical education had a positive effect in all three areas of Bloom's Taxonomy ( 28 ). A review of the studies in the field of educational software is presented in Table 3. Due to the fact that most dentists work independently in their office after graduation; it seems that a software that dentists would use is essential ( 14 ). According to the satisfaction results of the students and professors in this study, it was found that most of them agreed that this software would also be used after graduation.

**Table 3:** A brief review of the studies in the field of educational software

Authors (year)	Country	Type of software	Participants	Evaluation Method	Main findings
Chang et al, 2012 ( 23 )	Botswana	Telemedicine pharmaceutical software	Post-graduate medical students (N=7)	Satisfaction assessment after 4 and 8 weeks	The software significantly affected the amount of medical information in patients' beds and the amount of learning at home
De Sena et al., 2013 ( 10 )	Brazil	Skin flap surgery software	Medical students (N=50)	Pre-test/ post-test	The skill score of the students who used the software was higher than the group who were trained in the traditional way
Khanal et al., 2014 ( 11 )	United States	Cardiopulmonary resuscitation virtual reality simulator	Medical students (N=148)	Pre-test/ post-test	The simulator was as effective as the traditional face-to-face method
Yoo et al., 2015 ( 24 )	South Korea	Cardiopulmonary evaluation software	Nursing students (N=22)	Pre-test/post-test	The application improved students' knowledge and clinical skills
Bullock et al., 2015 ( 29 )	United Kingdom	Mobile software	Medical students (N=125)	questionnaire	Significant decrease in the use of printed copies and increase in the satisfaction among students was observed
Fernandez et al., 2016 ( 30 )	Spain	Shoulder examination skill software	Physiotherapy student (N=49)	Post-test	The OSCE test score of the intervention group was higher than the control group
Briz-Ponce, et al., 2016 ( 31 )	Spain	Anatomy software	Medical students (N=30)	Pre-test/post-test	The intervention group performed better than the control group
Kim et al., 2017 ( 32 )	South Korea	Removing neonatal airway obstruction skill software	Nursing students (N=73)	Pre-test/ post-test	Satisfaction level and skill scores were higher in the intervention group compared to the control group
Deshpande et al., 2017 ( 13 )	India	Dentures treatment planning software	Dental students (N=120)	5-point Likert questionnaire	The software improved clinical reasoning and decision making skills
Mladenovic et al., 2020 ( 27 )	Serbia	Dental traumatic injury software	Dental students (N=31)	4-point Likert questionnaire	More than 90% of students were satisfied and better training during the corona period was observed
Nasiri et al., 2014 ( 33 )	Iran	Head and neck anatomy software	Medical students (N=62)	Pre-test/post-test	Mobile based education promotes anatomy learning Compared to the group that was trained to give lectures
Babazadeh et al., 2016 ( 5 )	Iran	Oral pathology software	Dental students (N=30)	Post-test	Smart- phone based education significantly affected students' final grades
Gilavand et al., 2016 ( 14 )	Iran	Systemic patients' considerations software	Dental students (N=60)	Pre-test/Post-test	The students using the software had a higher awareness about the dental treatment of patients with systemic diseases compared to the control group

In general, the content will be memorized well due to the continuous repetition, and a software based on mobile phones with the possibility of access at anytime and anywhere and continuous repetition improves training and learning ( 14 ). A large sample size including the students of different levels with different experiences and knowledge gives us a better evaluation of the applicability of this software.

## Conclusion

According to the results of this study, the designed smartphone software improved the students' knowledge and increased their grades. The designed comprehensive software in various fields of dentistry satisfied the students and the professors who used this software; and the students' scores increased after using it. The use of a comprehensive software is effective in improving the students' scientific learning, scientific knowledge, and treatment planning.
